# Keyword Index for Volume 94

**DOI:** 10.1038/sj.bjc.6603218

**Published:** 2006-06-13

^131^I-tositumomab 1770

3D reconstruction 1176

5-fluorouracil 1287, 1809

5-fluorouracil/leucovorin 1122

5-FU adjuvant chemotherapy 1833

5-FU/cisplatin 281

*α*5*β*1 integrin 1621

*α*v*β*3 integrin 1621

A870D 928

AAV 1913

acidosis 884

acute childhood leukaemia 161

acute leukaemia 200

ADAM 724

ADAMTS 724

ADAMTS-8 1186

ADCC 259

adeno-associated virus 1913

adenocarcinoma 171, 896, 1460

adenomatous polyps 1718

adherence 507

adhesion 1311

adipocytokines 1221

adiponectin 156, 1221

adjuvant 492, 1122

adjuvant anthracyclines 1233

adjuvant therapy 30

adjuvant treatment 460

adoptive immunotherapy 1478

adrenomedullin 1

advanced and metastatic pancreatic cancer 481

advanced colorectal cancer 792, 964

advanced gastric cancer 959, 1130, 1803

aerobic glycolysis 578

aetiology 879

age and cancer 18

age at death 152

age-adjusted survival 446

ageing 593

AGR2 immunocytochemistry 1057

Akt 247, 398, 1906

albumin 227, 637, 1833

alcohol 757

ALL 1918

all-*trans*-retinoic acid (ATRA) 513

AML 323

AMN107 1765

anaemia 947

aneuploidy 891

aneusomy 891

anger 1245

angiogenesis 1, 101, 524, 552, 654, 1176, 1186, 1300, 1621, 1650

angiogenic factors 524

angiostatic 1845

angiotensin II 552

anthracycline pretreated 1615

antidepressant agents 1071

antihypertensive drugs 752

antiproteolytic drug 941

antithrombotic prophylaxis 189

antitumour immunity 391

anxiety 1093, 1253

apoptosis 13, 108, 253, 776, 1412, 1436, 1592

arachidonic acid 842

ARF 1555

ARF-BP1 1555

aromatase inhibitors 460, 1051, 1789

array CGH 1927

arsenic trioxide 398

AT_1_ receptor 552

ATM gene 1537

ATN-161 1621

audit 486

autoantibodies 1066

autocrine loop 1180

Axl 1446

azolyl retinoids 513

*β*-catenin 686, 1672

basal-cell carcinoma 743

basic anticancer drugs 863

*BCR-ABL* 1765

behavioural problems 43

benzoporphyrin derivative 391

bevacizumab 614

bexarotene 654

bFGF 1627

biliary disease 1751

bioactivation 1226

biochemical changes 1460

biomarkers 717, 1087, 1420, 1592, 1898

birth cohort 152

birth outcome 142

birth weight 1734, 1738

bladder cancer 1465, 1703

blood vessel density 1580

blood vessel invasion 1643

BMP-2 436

BMS-188797 79

body height 740

body mass index 740

bone loss 30

bone marrow 842

bone resorption 1496

borderline ovarian tumour 1586

boric acid 884

Borrelia burgdorferi 879

brain metastases 1777

brain tumours 1186, 1428

*BRCA* 814

*BRCA1* 308, 407

BRCA2 407

breast 253, 1333

breast cancer 8, 13, 30, 36, 43, 142, 152, 231, 239, 247, 299, 308, 333, 346, 358, 460, 469, 473, 524, 532, 540, 548, 672, 828, 1051, 1071, 1144, 1154, 1237, 1245, 1492, 1610, 1615, 1637, 1643, 1697, 1734, 1745, 1777, 1874, 1921

breast cancer risk 1537

breast cancer screening 147

breast cancer survival 147

breast epithelium 1021

breast neoplasms 427, 681

cachexia 731

CAEBV infection 599

CAM5.2 293

cancer distress 507

cancer genome 1927

cancer mortality 1079

cancer pain 1559

cancer stem cells 710

cancer survival 446

cancer therapy 941

cancer-specific survival 781

cancer/testis antigen 710, 1864

capecitabine 74, 959, 964, 969, 976, 1122, 1281, 1407

carboplatin 55, 62, 74, 1267, 1580

carcinoma 1472

cardiac toxicity 1016

case–case–control 743

case-control studies 752, 1071

catabolism 513

CCI-779 614

*CCND1* 928

CCNL1 1041

CD13 1627

CD4 cell counts 1000

CD4^+^ T cells 275

CD8^+^ T cells 275

cell cycle 93, 1045

cell cycle arrest 532

cell cycle control 184

cellular proliferation 776

central venous catheters 189, 200

cervical cancer 1045, 1683, 1913

cervical intraepithelial neoplasia 1253

cervical neoplasms 171, 1678

cervix 115, 1690

cetuximab 792

CGH 333

chemobyl 1472

chemokines 1029

chemoprevention 407

chemoradiation 363

chemoradiotherapy 351, 1375, 1389

chemosensitivity 203

chemotherapy 18, 51, 427, 524, 692, 785, 828, 1011, 1087, 1267, 1369, 1389, 1407, 1572, 1837

chemotherapy activity 1099

childhood leukaemia 156, 763, 1342

chloroquine 863

CHOP 806

chromatin 179

chromosomal damage 308

chromosome 11q23.2 1524

chromosome 3q 1041

chromosome instability 1485

circulating DNA 1492

circulating endothelial cells 524

circulating tumour cells 8

circumferential resection margin 351

cisplatin 79, 1375

c-jun N-terminal kinase 532

c-kit 1874

CKMT1 698

CL 1

clear cell 642

clear cell carcinoma 1369

clinical response 1051

clinical trial 609, 1136, 1237, 1267, 1420, 1803

c-MYC 1658

cognitive dysfunction 828

cohort study 142, 171, 1339, 1533

collagen XVIII 1066

colorectal adenoma 928

colorectal cancer 69, 128, 311, 586, 798, 928, 1116, 1122, 1136, 1287, 1300, 1320, 1412, 1478, 1672, 1710, 1816, 1823, 1833, 1898

colorectal carcinogenesis 922, 1718

colorectal liver metastases 982

colorectal polyp 311

combination chemotherapy 1130, 1287

combination effects 1837

combination therapy 69, 1604

combined modality treatment 1389

communication 208

concurrent radiotherapy 625

concurrent therapy 1375

conditionally replicative adenovirus 1837

conjunctiva squamous cell carcinoma 450

conjunctival intraepithelial neoplasia 450

conservative surgery 1586

continual reassessment method 609

contrast enhancement 427

correlation 1226

correlative markers 1136

cost-effectiveness 492, 1122

country of birth 1079

COX-2 1154, 1300

C-reactive protein 227, 1568, 1833

CRLR 1

cutaneous T-cell lymphoma 879

CXCL6 1936

CXCR1 1936

CXCR2 1936

cyclin A 1697

cyclin D 928

cyclin E 1045

cyclin L1 1041

cyclo-oxygenase (COX) 253, 346

cyclooxygenase-2 1718

cyclophosphamide 1226

CYP26 513

cystectomy 1586

cystoplasty 891

cytokeratin-19 672, 1164

cytokines 1412

cytology 1170, 1690

D2-40 293, 1643

dairy fat 165

dairy products 165

dasatinib 1765

database 318

dbpC/contrin 710

DCE–MRI 1420

DCIS 253

DDD 1204

delay 955, 1272

denaturing HPLC 268

denial (minimising) 1245

depressed neoplasia 311

depression 372

depressive disorder 1093

dermcidin 1663

dexamethasone 1011

diagnosis 904, 1272

diagnostics 1492

differential diagnosis 1726

differentiation 1637

diffusion study 427

disseminated epithelial cells 672

DJ-1 620

DNA damage 1194, 1942

DNA deletion 1887

DNA methylation 179, 1087

DNA quantification 1492

DNA repair 1194

DNA-PK 1683

*DNMT3b* 593

docetaxel 55, 62, 1233, 1375, 1402, 1803

dorsal skinfold chamber model 101

dose dense 1237

dose escalation 609

dose-finding studies 609

downstaging 1099

doxorubicin 1797

DR5 398

drug design 941

drug resistance 1087

DU-145 884

Dukes stage 1833

dynamic contrast-enhanced magnetic resonance imaging 1420

dysplasia 1170

EBV DNA microarray 599

E-cadherin 661, 1326, 1816

education 152

efaproxiral 1777

EGFR 85, 771, 896, 1136, 1144, 1703

EGGCT 820

eIF4E binding proteins 195

eIF4F 195

elderly 806, 969

electromagnetic fields 161

EMD 72000 1293

emotional problems 43

EMR 407

endocrine therapy 30, 828, 1789

endoplasmic reticulum stress response 407

endosomal pH 863

endostatin 1066

endothelial cells 1, 1428, 1845

England and Wales 1079

EP receptors 1154

Ep-CAM 128

epidemiology 142, 171, 737, 1533, 1690, 1751

epidermal growth factor 1703

epidermal growth factor receptor 184, 1293

epigenetics 179, 914, 1087

epirubicin 74, 1233, 1237, 1263, 1572

epithelial–mesenchymal transition 661

epoetin alfa 947

Epstein–Barr virus 599, 1504

*ERα* 593

ER*α*-positive breast cancer 1057

ER*β* varient 1333

ErbB 1703

erbB2 247

erlotinib 614, 1136

esophageal cancer 1281

estimated dose 1342

etoposide 1263

Ewing's sarcoma 22

exposure 1226

expressed emotion 1245

extracellular matrix 1311

extragonadal germ cell cancer 820

family health 499

family history 231

Fas-associated death domain 532

fertility 1007

FFPE archive 333

FGFR4 Arg388 polymorphism 1879

FGFR4 protein expression 1879

fibronectin 1311

fibrosarcoma cells 854

filgrastim 1237

first-line 959, 1789

FISH 1452, 1472

flat adenoma*BRAF* mutation 311

FLIP 398

fluorescence imaging 391

fluorescence *in-situ* hybridisation 891

fluorouracil 1107

folate 964

FOLFOXIRI 798

folic acid 1942

fractional polynomials 1785

FRAT1 686

frizzled related protein 922

fulvestrant 1021

GAGE 1864

G-CSF 806

gastric cancer 136, 281, 1204, 1281, 1402, 1407

gastrin 1107

gastrointestinal tumour 1180

gastro-oesophageal cancer 637, 1568

GCP-2 1936

gefitinib 85, 631, 1599

gelatinases 1035

gemcitabine 62, 1293, 1572, 1575, 1797

gemcitabine plus oxaliplatin 481

gemcitabine triphosphate 1797

gene expression 121

gene expression analysis 1927

general practice 1116

genetic polymorphism 203

genistein 407

germ cell tumours 1231

gestational trophoblastic disease 51

glioblastoma multiform 108

glioma 752, 1186, 1853

glutathione S-transferase P1 281

grade 781

*GRAF* 323

green fluorescent protein 391

Groucho/TLE 771

growth arrest 1637

growth regulatory gene 1524

*GSTP1* 473

HAART 1000

haemoglobin 947

haplotypes 1537

HCC 737

HCT116 1326

HCV 737

head and neck 1041

head and neck cancer 631, 647, 692, 955, 1516

head and neck squamous cell carcinoma 1164

health 1942

hepatocellular carcinoma 287

HER 1703

HER2 85, 1144

HER3 1144

hereditary cancer 814

high response 1575

high-dose chemotherapy 1016

high-risk populations 1537

histamine dihydrochloride, oxidative stress 218

histone deacetylase inhibitor 1436

histones 179

HIV 1000

hnRNP K protein 586

HNSCC 1041

Hodgkin's lymphoma 469, 1007

homologous recombination 1326

hormone replacement therapy 136

hPTTG1 1672

HPV 1913

h*TERT* 870, 1452

human immunodeficiency virus 450, 1504

human kallikrein 14 540

human papillomavirus 171, 1690, 1913

human pituitary tumour transforming gene 1672

human tumour specimens 1516

hypoxia 115, 121

hypoxia-inducible factor 115

IGFALS 299

IGFBP-1 299

IGFBP-3 299

IGF-I 299

imatinib-resistance 1765

immune suppression 1412

immunohistochemistry 108, 275, 532, 540, 710, 904, 1678, 1864, 1894, 1906

immunoprecipitation 548

immunotherapy 1029, 1785, 1864

*in utero* exposure 1734

incidence 1342, 1751

indole-3-carbinol (I3C) 407

inducible nitric oxide synthase 184

infant 1510

inflammation 731, 1465

inflammation-mediated tumour progression 854

inhibitors 513, 1355

insulin resistance 1221

insulin-like growth factor receptors 465

insulin-like growth factors 465

interferon 1465

interferon-alpha 492

interferon-*γ* 1845

interleukin-2 218

invasion 1186, 1627

invasiveness 1428

investigations 1116

involved field 625

IRESSA 1599

irinotecan 55, 792, 976, 1130, 1267, 1281, 1287, 1402, 1407

irradiation 308

ISG15 1465

isolated tumour cells 293

JACC study 737

Japanese 737

Jewish breast cancer patients 1537

kallikrein 540

Kaposi's sarcoma 1000, 1504

Kaposi's sarcoma-associated herpesvirus 1504

keratinocytes 85

K-ras 896

lactose 165

laminin 1311

laparoscopic ultrasound 213

laparoscopy 213

laser capture microdissection 569, 1452

late effects 469

late recurrence 820

late relapse 820

LDH 1231

leucovorin 1287

leukaemia 156, 161, 398, 1738, 1918

limited stage 625

liposomal doxorubicin 1615

liver 1663

liver disease 1751

longitudinal studies 1093

long-term follow-up 1770

long-term survivor 1099

loss of heterozygosity (LOH) 1485

lung 724

lung cancer 18, 128, 896, 1029, 1176, 1485, 1936

lung metastasis 51

lyme disease 879

lymph node metastasis 1369

lymph nodes 1164

lymph vessel invasion 1643

lymphangiogenesis 1154, 1650

lymphatic density 1650

lymphatic vessel 1355

lymphoblastic leukaemia 161

M30-ELISA 1592

macrophage 101

MAGE-A1 1864

magnetic resonance 427

magnetic resonance imaging 351

MALDI-TOF-mass spectrometry 698

malignancy 189

malignant spinal cord compression 486

malignant transformation 195

mammaglobin 672, 681

mammary cell lines 661

mantle radiotherapy 469

MAPK pathways 311

mass screening 499, 1253, 1510

mass spectrometry 1898

maternal age 1738

maternal weight 1738

mathematical model 93

matuzumab 1293

maximum tolerated dose 609

MCF-7 1637

MDA-MB-231 1637

Mdm2 1555, 1683

*MDR1* 473

MDS 323

melanoma 492, 743, 835, 1496, 1627, 1879

menstrual cycle 1690

mercaptopurine 93

mesothelin 436

metallothionein 835

metastasis 13, 654, 785, 798, 842, 854, 1936

metastatic breast cancer 227, 1016, 1233, 1604, 1789

metastatic colorectal cancer 969

metastatic pancreatic cancer 1575

methodology 1559

methylation 561, 914

methylation-specific PCR 1492

M-FISH 308

microarray 436, 904

micrometastasis 293, 681, 1164

microRNAs 776

microsatellite instability 1485

microvessel density 1823, 1879

migration 884, 1311

milk 165

minichromosome maintenance proteins 1170

minisatellite instability 1485

mitochondrial DNA 692, 1887

mitochondrial DNA mutations 268

mitochondrial encephalomyopathy with lactic acidosis and stroke-like episodes 268

MMP 569

MMP-7 1035

MMP-8 1035

MMP-9 1035

MoA 259

models of viral transmission 1533

modified nucleosides 1726

monoclonal antibodies 128, 465

Moran's I 763

mouse mammary tumour virus (MMTV) 548

mouth neoplasm 647

MR 427

*MRP1* 473

mTOR 195

mucinous 904

multidisciplinary team 351

multiple primary cancers 1745

multivariate analysis 1586, 1853

mutation 318, 692, 896, 1918

*MYCN* 1510

mycosis fungoides 879

Myeov 1204

*MYOD* 593

naive patients 1000

nasal NK/T-cell lymphoma 599

nausea 1011

neck dissection 1164

neoadjuvant chemoradiotherapy 976

neoadjuvant chemotherapy 358, 363

neoadjuvant therapy 1051

nephrectomy 781

Net1 1204

neuroblastoma 13, 1510, 1845

NF-*κ*B 1436

nilotinib 1765

non-Hodgkin's lymphoma 806, 1339, 1504, 1770

non-seminoma 820

non-small-cell lung cancer 275, 1375, 1383, 1599, 1927

Notch 771

novel gene 1524

NSAIDS 346

nuclear sites 1342

nucleostemin 1658

NY-BR-1 681

obesity 1221

oesophageal adenocarcinoma 136

oesophageal carcinoma 1389

oesophagus 203, 1460

oestradiol 1021

oestrogen receptor 1333

oestrogens 136, 1734

Omega 3 842

Omega 6 842

omeprazole 863

oncogene 776

opioids 1559

oral cavity 1170

oral cavity cancers 1580

oral squamous cell carcinoma 561, 698, 717

orally available superoxide dismutase 854

osteoclast 1496

osteosarcoma 22, 1837

outcomes 36, 1361

ovarian cancer 62, 74, 436, 552, 686, 757, 814, 904, 914, 1650

ovarian cryopreservation 1007

ovarian neoplasms 165

ovary 55, 947, 1369

oxaliplatin 69, 785, 959, 969, 1809

oxidative phosphorylation 268

p110*α* 455

p21 1683

p300 1326

p53 1555, 1683

paclitaxel 1237, 1263, 1369, 1894

pAKT 1678

palliation 631

pancreatic cancer 213, 785, 1107, 1293, 1572

papillary 1472

papillary serous 642

papillomavirus 450

Parkinson's disease 620

Parthenolide 1436

pathological complete response 358

patient survival 1057

patterns of care 22

pentose phosphate pathway (PPP) 578

performance status 781

peripheral blood lymphocytes 1194

pertuzumab 85

PG-dependent gene expression 1718

pharmacodiagnostic marker 578

pharmacoeconomics 1122

pharmaco-epidemiology 752

pharmacogenetics 281

pharmacological interventions 372

phase 1 trial 609, 1621

phase I/II 1803

phase II study 792, 1136, 1287, 1383, 1395, 1575

phosphorylation 532, 586, 870

photodynamic therapy 391

physician-patient relations 208

PI3K 455, 1678

PIK3CA 455

PKC isoenzymes 870

placebo 1107

PMitCEBO 806

Poly(A) binding protein- interacting protein 2 1516

poly(ADP-ribosyl)ation 341

polymerase chain reaction 879

polymorphism 593, 928

pooled analysis 757, 1789

poor prognosis 717

population-based 231

population-based study 1339

postmenopausal 1789

postmenopausal hormone replacement therapy 1339

Potthoff–Whittinghill method 763

pregnancy weight gain 1738

preoperative radiotherapy 1809

primary cell cultures 1837

primary systemic therapy 259

prognosis 8, 208, 358, 473, 552, 692, 731, 1785, 1816, 1874, 1906

prognostic factors 275, 835, 1823

prognostic marker 540

prognostic score 227

prognostic value 1516

proliferation 1021, 1051, 1412

promoter methylation 323, 593, 661

prophylactic surgery 814

prospective study 740

prostaglandins 346, 1718

prostate cancer 128, 507, 740, 842, 884, 1361, 1592, 1906

prostate-specific antigen 499

prostatic neoplasms 499, 1093

protein kinase C 1436

proteinases 724

proteome 287

proteomics 717, 1853, 1898

PSA recurrence 1906

psychological factors 1253

psychological morbidity 507

psychotherapeutic interventions 372

PTEN 247, 620

PU.1 1918

pyrosequencing 561

QC 333

QPCR 1452

quality of life 492, 947, 1093, 1116

quantitative RT–PCR 569

questionnaires 1253

radiation resistance 1678

radiation sensitiser 1777

radiation therapy 1375, 1580

radiation-induced fibrosarcoma 391

radioimmunotherapy 1770

radiosensitivity 1683

raltitrexed 785, 1809

Raman spectroscopy 1460

RAMBAs 513

randomised trials 1233

RANKL 1496

rapamycin 195

rat 1853

*RB1* 1921

RCC 1658

real-time PCR 731, 1164, 1887

receptor tyrosine kinase 184, 1446

rectal cancer 351, 363, 976, 1809

recurrence 253

referral 955

relapse 74, 1231

renal cell carcinoma 218, 268, 781, 1217, 1785

renal tissues 1658

repression 1245

resection 1568

residual ductal carcinoma *in situ* (DCIS) 358

residual invasive carcinoma 358

retinoid X receptor 654

retroperitoneal lymph node dissection 820

risk 752, 1339, 1785

RNA editing 586

RNA Interference 1300

RSR13 1777

RT–PCR 473, 672, 681

S-1 1130, 1575, 1803

safety 964

salvage therapy 785

sarcoma 1180

scan statistic 763

SCF 1180

SCLC 625, 1263

screening 507, 896, 1093, 1170, 1690

screening as independent prognostic factor 147

second line chemotherapy 481

SEER 1745

SELDI 287, 1853, 1898

selective COX-2 inhibitor 346

seminoma 820

senescence 884

sentinel node 1478

sequencing schedules 460

sequential therapy 62

serine protease 540

seroprevalence 548

serous tumour 1586

serum 287

serum proteome 1898

side effects 1011

signal transduction 771

single nucleotide polymorphisms 299, 921

single-cell gel electrophoresis 1194

skin cancer 743, 1446, 1887

skin rash 85

Slug 1816

small-cell lung cancer 1267, 1927

small-molecule EGFR tyrosine kinase inhibitors 1604

smoking 896

SNP 1537

soft tissue sarcoma 1797

somatic 318

somatic mutations 455

sorafenib 614, 1217

space–time analyses 763

sparse data 446

spatial heterogeneity 763

specialisation 36

spectroscopy 427

squamous cell carcinoma 171, 203, 743, 1041, 1170

Src kinase 1710

stage IIIA NSCLC 1099

staging 213

stathmin 717

Staurosporine 1436

steroid receptors 1021, 1051

stomach cancer 128

sun exposure 743

sunitinib 614, 1217

surgery 982, 1116

surveillance 814, 1231

survival 22, 36, 227, 231, 239, 637, 642, 647, 982, 1245, 1412, 1568, 1663, 1823, 1833, 1879

survival analysis 208

survivin 108, 253

susceptibility to chemotherapy 1894

systematic review 372, 982, 1272, 1559

systemic inflammatory response 227, 781

tamoxifen 1021

tankyrase 341

target validation 941

targeted intra-arterial chemotherapy 1580

targeted therapy 1144

tau 1894

taxanes 55, 79, 1375, 1604

telomerase 341, 870, 1452

telomeres 341

testicular cancer 820

testis 1864

TFG*β* pathway 661

therapeutic monitoring 8

thrombomodulin 1320

thrombosis 189

thymidine phosphorylase 115

thymidylate synthase 121, 281

thyroid 1472

time trends 152

TIMP 1035

tissue inhibitor of metalloproteinases 1035

tissue microarray 686, 1697

tissue penetration 863

tissue remodelling 1428

TNM stage 1568

TNM stage C-reactive protein 637

tobacco 692

toxicity 609

trabectedin 1610

TRAIL 398

transcription factor 1918

transcriptional regulation 184, 771

transfection 698

transforming growth factor-beta1 239

transfusion 647

transitional cell carcinoma (TCC) of the bladder 1395

transketolase (TKT) 578

transketolase-like-1 (TKTL1) 578

translational control 195

trastuzumab 247, 259, 1016, 1144

treatment 231, 1592

triple-agent therapy 62

truth disclosure 208

tumour 1, 1472, 1845

tumour budding 293

tumour marker 8, 1726

tumour reactive lymphocytes 1478

tumour regression grade (TRG) 1809

tumour stage 781

tumour suppression 620

tumour suppressor 776

tumour thickness 1879

twist-1 13

two-dimensional electrophoresis 698

tyrosine kinase inhibitors 465

ubiquitin-like protein 1465

UCN-01 1436

UFT® 69

Uganda 450

ultraviolet radiation 1887

upper gastrointestinal 1272

uracil misincorporation 1942

urine 1726

urothelial cancer 569

uterine 642

valproic acid 1436

vascular density 1650

vascular endothelial growth factor (VEGF) 101, 552, 724, 1217, 1710

vascular endothelial growth factor-A 1516

VEGF expression 1823

VEGF-C 1154, 1355

VEGF-D 1355

VEGFR-1 1710

VEGFR-3 1355

veno-occlusive disease 1226

venous thromboembolisms 200

vinflunine 1383, 1395

viral load 1000

virotherapy 1837

vomiting 1011

von Hippel–Lindau 614

Warburg effect 578

WEB-2086 1637

Western immunoblot 548

whole-brain radiation therapy 1777

Wilms tumour 1524

Wnt pathway 686

Wnt-signalling 922, 1672

XELOX 969

Yondelis 1610

zinc chelation 941

